# Point of care transthoracic echocardiography for the prediction of post – spinal anesthesia hypotension in elderly patients with cardiac diseases and left ventricular dysfunction: Inferior vena cava and post-spinal anesthesia hypotension in elderly patients

**DOI:** 10.1007/s10877-023-00981-y

**Published:** 2023-02-21

**Authors:** Nefeli Moschovaki, Theodosios Saranteas, Elen Spiliotaki, Dimitrios Giannoulis, Dimitrios Anagnostopoulos, Christina Talliou, Orestis Milionis, Panagiotis Briassoulis, Konstantinos Katogiannis, Thomas Papadimos

**Affiliations:** 1 Second Department of Anesthesiology, Medical School, National and Kapodistrian University of Athens, 23 Agnoston Iroon str, 15349 Athens, EU Greece; 2 Second Department of Cardiology, Medical School, National and Kapodistrian University of Athens, Athens, EU Greece; 3 Wexner Medical Center, Ohio State University, Columbus, OH USA

**Keywords:** Post - spinal anesthesia hypotension, Echocardiography, Inferior vena cava

## Abstract

In elderly patients with cardiac diseases, changes in cardiovascular physiology diminish cardiovascular reserve and predispose to hemodynamic instability after spinal anesthesia; hence, such patients could be at risk of postoperative complications. Additionally, transthoracic echocardiography (TTE) is used in clinical practice to evaluate cardiovascular hemodynamics. Therefore, we hypothesized that echocardiographic measurements could display significant diagnostic power in the prediction of post - spinal anesthesia hypotension in elderly patients with cardiac diseases and reduced left ventricular ejection fraction (LV-EF). Therefore, sixty-one elderly orthopedic-trauma patients were recruited. Prior to spinal anesthesia a TTE examination was performed. The LV-EF, the stroke volume index (SVI), the peripheral vascular resistance (PVR), the LV filling pressures (E/Em ratio), the right ventricular function [tricuspid annular plane systolic excursion (TAPSE), tricuspid annular systolic velocity (TASV) and fractional area change (FAC)], as well as inferior vena cava (IVC) measurements, such as IVCCI (collapsibility index of the IVC) and dIVCmax (maximum diameter of IVC)-to-IVCCI ratio were assessed. Twenty-six out of sixty-one patients manifested hypotension. Preoperative dIVCmax-to-IVCCI ratio demonstrated the greatest performance amongst echocardiographic indices in predicting post - spinal anesthesia hypotension. The dIVCmax-to-IVCCI ratio < 48 had significantly higher diagnostic power than IVCCI > 0.28, FAC > 42, E/Em ratio < 9 and SVI < 32 (receiver operator characteristic curve analysis). The gray zone for the dIVCmax-to-IVCCI ratio (40–49) showed the lowest number of inconclusive measurements among echocardiographic variables. The preoperative dIVCmax-to-IVCCI ratio could be a reliable echocardiographic index to predict post - spinal anesthesia hypotension in elderly patients with left ventricular dysfunction.

## Introduction

The incidence of chronic heart failure has an increasing prevalence, rising to almost 10% of people older than 80 years old [1, 2]. The elderly population is also at high risk of dehydration primarily because of a decreased thirst sensation [3]. At the other edge of the spectrum, overhydration is also a frequent medical condition pertaining to heart failure as well as to an overzealous iatrogenic provision of fluids [1, 2]. In addition, changes in cardiovascular physiology in elderly patients with cardiac diseases reduce cardiovascular reserve and predispose to significant hemodynamic instability. Under these circumstances, sympathetic nervous system activity increases in the elderly population and, particularly, in those with congestive heart failure. Therefore, such patients could be at risk of greater decreases in systemic vascular resistance (SVR) and blood pressure than younger individuals [4, 5]. In this context, spinal anesthesia may cause either severe intraoperative hypotension due to sympathectomy, or overhydration if an excessive amount of fluids is given during volume replacement. Both conditions can lead to severe perioperative complications [2–8].

Point of care transthoracic echocardiography (TTE) is featured prominently in clinical practice to evaluate cardiovascular hemodynamics [9–11]. In fact, there are numerous echocardiographic indices, as well recommendations regarding the optimal thresholds of echocardiographic measurements in various clinical scenarios and contexts [9–12]. Inferior vena cava (IVC) ultrasound measurements have shown high specificity in the diagnosis of acute dyspnea of cardiac origin [9]. In Orso et al. study [12], the blood urea nitrogen/creatinine ratio has been referred as the reference value in foreseeing elderly patients’ dehydration; a good correlation between IVC indices and this ratio was found. Furthermore, in elderly patients, the IVC diameter at expiration, as well as lactate and pH values before spinal anesthesia, were effective in predicting post-spinal hypotension [10]. Recently, the dIVCmax-to-IVCCI ratio has shown significant diagnostic power to predict hemodynamic instability, secondary to spinal anesthesia, in elderly patients with normal LV-EF [11]. However, its value in patients with low LV-EF has not been investigated.

In the light of these findings, we hypothesized that echocardiographic measurements could display significant diagnostic power in the prediction of hypotension after spinal anesthesia in patients with cardiac diseases. For this reason, we prospectively evaluated echocardiographic indices of the LV and the right ventricle (RV), as well as of the IVC prior to spinal anesthesia in elderly patients with proximal femur fractures who had low LV-EF.

## Materials and methods

This was an open cohort, prospective, single center, study. It was approved by the Institutional Ethics Committee of Attikon University Hospital of Athens, Greece, EU (No: 333, 9-12-2017, enrolment period: from August 1, 2019, until April 29, 2022), registered at ClinicalTrials.gov registry, (NCT: 0400188), and revisited in 5-11-2021.

### Patients’ initial assessment and screening

Elderly patients (age > 70 years) scheduled for proximal femur fracture repair under spinal anesthesia were recruited. Informed consent was obtained from the patients or their surrogates. All patients underwent a preoperative anesthetic evaluation by the anesthesiology department as well as a cardiac evaluation by the cardiology department. Based on the cardiology consultation, we identified those patients who were American Heart Association/American College of Cardiology (AHA/ACC) stage B or C [13] whose cardiac disease status was compensated. Patients who fulfilled the above criteria had a preoperative TTE examination performed prior to spinal anesthesia (in the operating theater).

### Definition of inclusion and exclusion criteria

Exclusion criteria included patients with tachycardia (heart rate > 100 beats/min), atrial fibrillation or left bundle branch block on ECG. Poor acoustic windows, pulmonary hypertension (peak tricuspid velocity > 3.4 m/sec), tricuspid/mitral/pulmonary valve regurgitation grade 3 or 4, severe aortic/mitral valve stenosis, or severe mitral annulus calcification on the preoperative echocardiogram (in the operating theater). Additionally, patients who had the maximum cephalad dermatomal extension of the spinal sensory block below T12 or arterial hypotension related to overt intraoperative bleeding (> 150 cc) were also excluded.

All patients included for analysis had an LV-EF between 35% and 50% and normal RV function indices [tricuspid annular plane systolic excursion (TAPSE) index > 16, tricuspid annular systolic velocity (TASV) > 10 cm/sec and fractional area change (FAC) > 35%)] [14, 15] on the preoperative echocardiogram (in the operating theater).

### Baseline blood pressure measurements

In all the participants, the medical treatment for hypertension and/or CHF (e.g., angiotensin converting enzyme inhibitor) except for beta blockers was discontinued the day of surgery.

Upon arrival in the operating room (preparation area), standard non-invasive monitoring (continuous ECG, non-invasive blood pressure measurements every three minutes and SPO_2_) was applied.

Non-invasive blood pressure measurements using a sphygmomanometer were taken in an upper extremity before radial artery cannulation. The mid-arm circumference was used to ascertain the appropriate brachial cuff size (M, M + or L). Three consecutive non-invasive MAP measurements were recorded, and their average was used as reference value. For invasive blood pressure measurements, a 20-gauge radial artery cannula was connected to a disposable pressure transducer (Edwards life sciences LLC, Irvine, California, USA) and continuously monitored. The arterial line was zeroed at the level of the right atrium. In addition, air bubbles were purged from the system prior to data collection, and the appropriate blood pressure waveform was determined using the “fast flush” test. Both invasive and non-invasive blood pressure data were recorded from the display of the bedside monitor (B 40, GE, health care, Helsinki, Finland).

### Preoperative echocardiographic examination

**Fig. 1:**
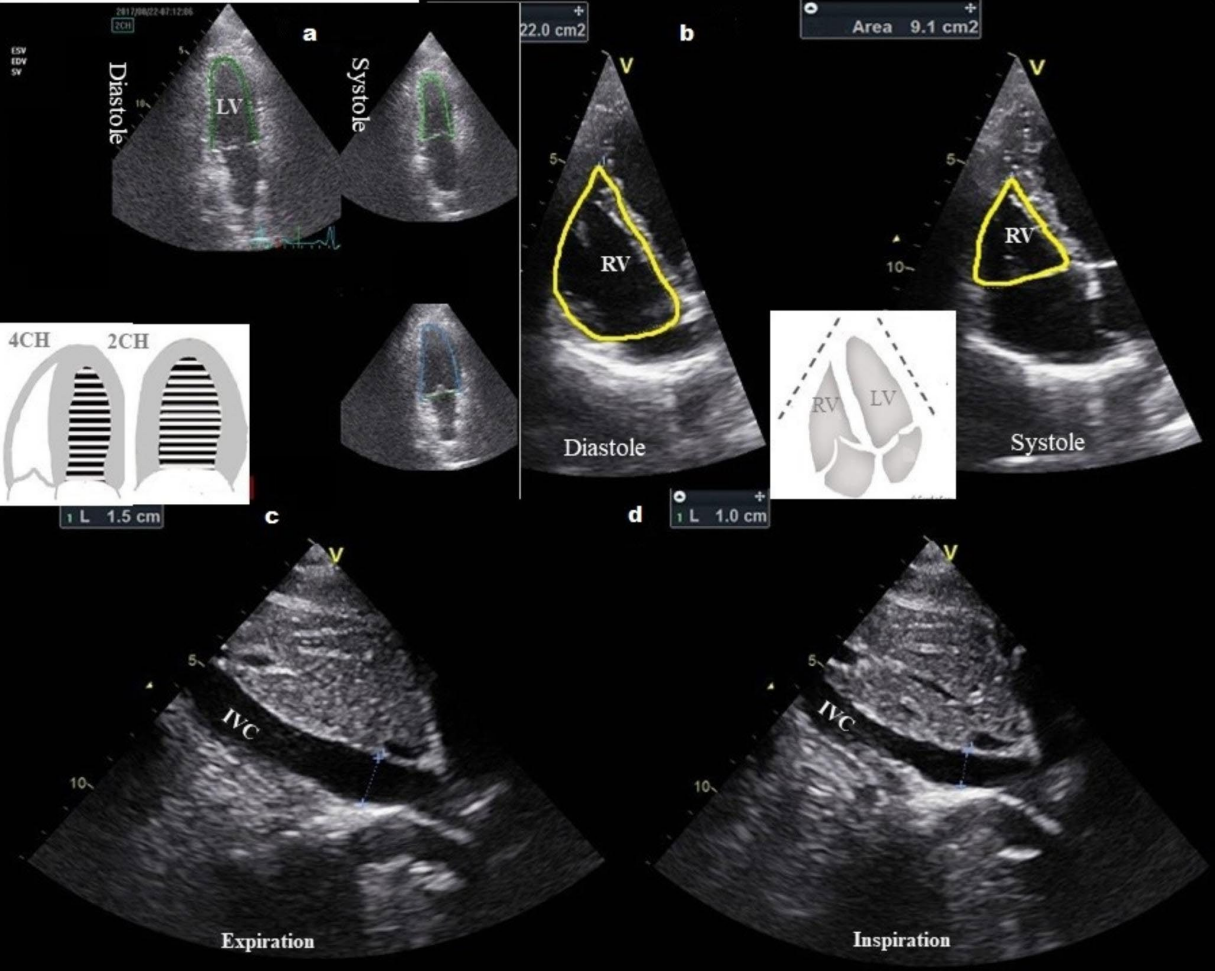
TTE-non-invasive hemodynamic monitoring of the heart. **a** Biplane Simpson’s method of disks with automated measurements of LV volumes for the calculation of stroke volume and ejection fraction of the LV. **b** RV focused view: From the 4 chambers view, the transducer was moved laterally and rotated until the maximum diameter of the base is visualized. To measure the Fractional Area Shortening, the endocardial borders of the RV are traced to assess the RV area both in diastole and systole. **c and d** Subcostal view of IVC at expiration and inspiration. 2CH = 2 chambers view, 4CH = 4 chambers view, RV = right ventricle, LV = left ventricle, IVC = inferior vena cava

After baseline MAP measurements and prior to spinal anesthesia induction, a TTE examination (Vivid T8, GE Healthcare, USA, equipped with 1.7-4 MHz phased array transducer) was performed, which included the following views: the parasternal long (LAX) and short axis (SAX), the apical 4-chamber (4CH) and 2-chamber (2CH) (Fig. 1a), the “RV-focused view” (Fig. 1b), as well as the apical 3-chamber (3CH) and the subcostal IVC (SUB-IVC) (Fig. 1c and d) [14, 15]. All views were saved for off-line analysis.

To assess the function of the right ventricle (RV), the fractional area change (FAC) (2-dimentional surrogate for RV-EF) was calculated from the “RV-focused view” (Fig. 1b) by using the following formula [14]: 100 × (RV-Area end-diastolic − RV-Area end-systolic) / RV-Area end-diastolic). Tricuspid regurgitation grading and velocity were assessed according to pertinent guidelines [15, 16]. The LV-EF was estimated by the Simpson’s method of discs by performing measurements of LV volumes in the 2CH and 4CH views (semi*-*automated EF quantification, Auto EF 2.1, Vivid T8, GE Helthcare, USA) (Fig. 1a). LV filling pressures were estimated by the E/Em ratio (E = peak velocity of mitral flow in early diastole, Em = the average of peak velocities in early diastole of lateral and septal mitral annulus) [11, 17]. Stroke volume (SV) and subsequent stroke volume index (SVI = SV/m^2^) of the LV were assessed using automated measurements of LV volumes, according to the formula SV = EDV-ESV, where EDV = end diastolic LV volume, and ESV = end systolic LV volume [16]. From these data, we derived values for the assessment of cardiac output (CO) = SV x HR, and SVR = MAP x 80/CO, (MAP = mean arterial blood pressure, HR = heart rate) [18]. The IVC measurements included the IVC maximum diameter at the end of expiration (dIVCmax), and inspiration (Fig. 1c and d), the IVCCI during spontaneous, quiet, breathing [(IVC maximal diameter – IVC minimal diameter)/IVC maximal diameter], as well as the ratio of dIVCmax-to-IVCCI [15]. In suboptimal images, ultrasound contrast agents were used (SonoVue: Bracco, Milan, Italy and Optison: GE, Healthcare, USA). The anesthesiologist who performed the echocardiographic examinations and all the subsequent measurements was certified by the European Association of Cardiovascular Imaging. All measurements were repeated twice by a single investigator, and their arithmetical mean was used in the analysis. The anesthesiologist analyzing the data was blinded to the patients’ intraoperative hemodynamic status.

### Spinal anesthesia protocol

As per departmental fasting guidelines, all patients were allowed to receive clear liquids and solid food until 2 and 8 h before induction to spinal anesthesia, respectively. No pre-spinal anesthesia fluid load was given.

Ultrasound-guided fascia iliaca or supra-inguinal fascia iliaca nerve blocks were performed before spinal anesthesia to prevent patients’ pain during lateral position placement. Ten to 20 mg of propofol were administered prior to nerve blocks. Additional sedation/analgesics (e.g., propofol or remifentanil infusion) were not administered during the operation.

Spinal anesthesia was introduced with a single intrathecal injection of ropivacaine (0.75% solution) ranging between 12 and 18 mg (average dose 15 mg) depending on patients’ age, frailty and BMI, using a 22- or 25-gauge needle. The intrathecal injection was performed in inter-spaces L2-L3, L3-L4 or L4-L5. After subarachnoid injections, all patients immediately returned to supine position, except for those undergoing hip hemi-arthroplasty. Spinal anesthesia was followed by a continuous intravenous infusion of 1.5 ml/kg/h of Lactate Ringer’s solution. Sensory block was assessed every 10 min after injection (pinprick test) until the end of surgery. The maximum cephalad dermatomal extent of the sensory block was measured.

### Intraoperative blood pressure measurements and patients’ follow up

After spinal anesthesia induction, continuous, invasive blood pressure measurements were taken and patients with MAP ≤ 65 mmHg, or with a reduction ≥ 25% of baseline pre-operative values, were considered hypotensive (time interval of low MAP: 30 s, assessment period of low MAP: from spinal anesthesia induction to the end of surgery). Once the patients experienced hypotension (fulfilling the above criteria) the patients were treated according to the attending’s anesthesiologist clinical judgment.

The length of stay (LOS) in the post – anesthesia care unit (PACU) was also estimated and defined as the time from the patients’ admission to the time of their discharge from the PACU [11]. Patients who were hemodynamically stable during the intraoperative period, but experienced hypotension in the PACU, were not considered in the statistical analysis.

### Statistical analysis

A pilot study of 18 patients revealed a detected area under the ROC curve (AUC) of 0.89 for dIVCmax-to-IVCCI, and for IVCCI 0.74, with rank correlation between the two assays being 0.88 in positive and 0.65 in negative cases (unpublished data). Thus, at least a sample of 40 patients would be required to achieve a power of 80% to detect significant difference (at a level of 0.05) between dIVCmax-to-IVCCI ratio and IVCCI (MedCalc Software, Mariakerke, Belgium) [19, 20]. To correct for possible dropouts and to improve analysis accuracy, a sample size of at least 50 patients was used.

Quantitative variables and proportions were compared with the student *t*-test or Mann-Whitney and chi-square tests, respectively. Normality was tested by using the Kolmogorov- Smirnov test. We assessed the area under the receiver operator characteristic curve (AUC-ROC) and the respective 95% confidence interval (95% CI) according to Hanley and McNeil [19, 20]. AUC-ROC curves were compared using the method of DeLong et al. [21]. The gray zone analysis was conducted using the Coste and Pouchot method [22]; it was applied to determine a range of echocardiographic measurements in which proper conclusions about the prediction of MAP response (hypotension or normotension) cannot be acquired [22, 23]: therefore, the measurements within the gray zone were considered inconclusive. Interobserver agreement for the echocardiographic parameters was done with Bland-Altman plots and the Lin’s concordance correlation coefficient (CCC) between two independent measurements in 25 randomly selected patients; assessment of CCC was based on the following scale: poor (CCC < 0.90), moderate (0.90 ≤ CCC < 0.95), substantial (0.95 ≤ CCC < 0.99), and almost perfect (CCC > 0.99). The results were expressed as percentage (%) or mean ± SD (standard deviation); a *p* value of *< 0.05* was considered statistically significant, (SPSS, 17.0, Chicago, IL; MedCalc Software, Mariakerke, Belgium).

## Results

### Patient data

**Fig. 2:**
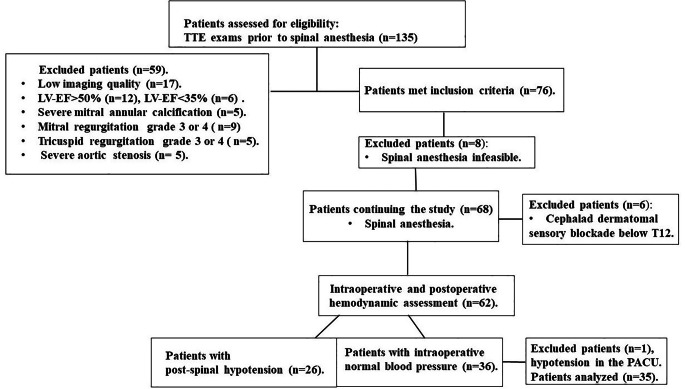
Flow diagram of patients’ cohort development. LV = left ventricle, RV = right ventricle, IVC = inferior vena cava, PACU = post-anesthesia care unit

The flow diagram of patients’ cohort is analyzed in Fig. 2. A total of 135 patients were enrolled in the study and had TTE examination in the operating theater (prior to spinal anesthesia induction). Fifty-nine patients were excluded, because they did not fulfil the echocardiographic inclusion criteria. Seventy-six patients were finally subjected to spinal anesthesia. In sixty-eight patients (30 men and 38 women; age 80 ± 9 years; BMI 28.9 ± 4 kg/m^2^; AHA/ACC stage (II/III) 23/45; American Society Association (ASA) physical status (PS) (II/III) 17/51; baseline MAP 88 ± 12 mmHg; baseline HR 77 ± 14 beats/min) the spinal anesthesia technique was successfully implemented. Six patients had cephalad dermatomal sensory blockade below T12 and were excluded from further evaluation. In the remaining 62 patients the cephalad extension of the anesthetic block was confirmed between the T12 and T2 dermatomes. Twenty six out of 62 patients (40%) manifested hypotension after spinal anesthesia (Fig. 2). Among the hemodynamic stable patients (n = 36), one who experienced hypotension in the PACU was excluded. No adverse events were found from performing echocardiography, invasive blood measurements and spinal anaesthesia technique.

### Univariate analysis

The univariate analysis of baseline clinical and echocardiographic variables in patients with normal blood pressure vs. spinal-induced hypotension is described in Tables 1 and 2. Any underlying cardiac condition that may have contributed to a reduced LV-EF in a patient is reported in Table 1.

**Table 1:** Patients’ characteristics

Patients’ characteristics	Normotensive patients, n = 35	Hypotensive patients, n = 26	*P*-Value
Age (years)	78.5 (70–91)	79 (70–97)	0.27
Gender (male/female)	16/19	12/14	0.8
BMI (kgr/m^2^)	30 (19–37)	31 (21–38)	0.48
ASA-PS (II/III)	10/25	7/19	0.75
Hb (g/dL)	11 (9-14.5)	12 (9–14)	0.2
Baseline Heart rate (pulse/min)	78 (91 − 50)	78 (45–99)	0.7
Baseline MAP (mmHg)	85 (67–118)	93 (69–120)	0.09
Time from spinal anesthesia induction to end of surgery (min)	80 (50–125)	89.5 (40–120)	0.49
Ropivacaine dose (mg)	15 (12-16.5)	15 (12–18)	0.1
Maximum cephalad extent of the sensory block	T7 (T12-T3)	T6 (T12-T2)	0.33
LOS in PACU (min)	80 (55–125)	95 (50–160)	0.037
***Type of orthopaedic surgery***			
Trans/intertrochanter fractures (supine position)	31/35	22/26	0.57
Hip hemiarthroplasty (lateral position)	4/35	4/26	0.64
***Type of cardiac disease***			
Hypertension	8/35	7/26	0.71
Coronary artery diseases	9/35	2/26	0.06
Hypertension + coronary artery diseases	17/35	17/26	0.16
Chemotherapy-induced cardiomyopathy	1/35	0/26	0.47

BMI = body mass index; ASA-PS = American Society of Anesthesiologists-physical status; Hb = hemoglobin; MAP = mean arterial *pressure; LOS = length of stay; PACU = post-anesthesia care unit.* Values are expressed as proportion or median (highest value-lowest value)

**Table 2:** Echocardiographic and hemodynamic data

Echocardiography and hemodynamics	Normotensive patients, n = 35	Hypotensive patients, n = 26	*P*-Value
Left ventricle (LV)			
LV-EF (%)	40.5 (30–50)	39 (30–50)	0.062
E/Em	10 (7–12)	9 (5–12)	0.03
SVI (ml/m^2^)	42 (28–65)	32.5 (17–46)	0.0014
SVR (dyn·s·cm^− 5^)	900 (670–1200)	1100 (770–1650)	0.02
Performance of contrast echocardiography	10/35	8/26	0.73
**Right ventricle (RV)**			
TAPSE index (mm)	21 (16–27)	22 (16–30)	0.6
TDI (tricuspid annulus)	12.2 (10–17)	13 (10–15)	0.8
FAC	41 (35–55)	45 (37–55)	0.032
dIVCmax-to-IVCCI	57 (35–140)	40 (12–52)	0.01
IVCCI (%)	0.25 (0.1–0.5)	0.3 (0.2–0.9)	0.025
DIVCmax	15 (9–25)	12.5 (4–23)	0.054
**TR grading**			
0	1/35	3/26	0.2
1+	27/35	17/26	0.3
2+	7/35	6/26	0.85
TR velocity (m/sec)	(n = 30) 2.8 (2.2–3.4)	(n = 19) 2.8 (2-3.2)	0.07

LV-EF = left ventricular ejection fraction; dIVCmax = maximum diameter of inferior vena cava; *IVCCI =* collapsibility index of inferior vena cava; SVI = stroke volume index; TDI = tissue doppler imaging; TAPSE = tricuspid annulus plane systolic excursion; E/Em = the ratio of peak velocity flow in early diastole to the average of peak velocities in early diastole of lateral and septal mitral annulus; SVR = systemic vascular resistance; TR = tricuspid regurgitation. Values are expressed as proportion or median (highest value-lowest value)

### Diagnostic performance of echocardiographic variables

**Fig. 3:**
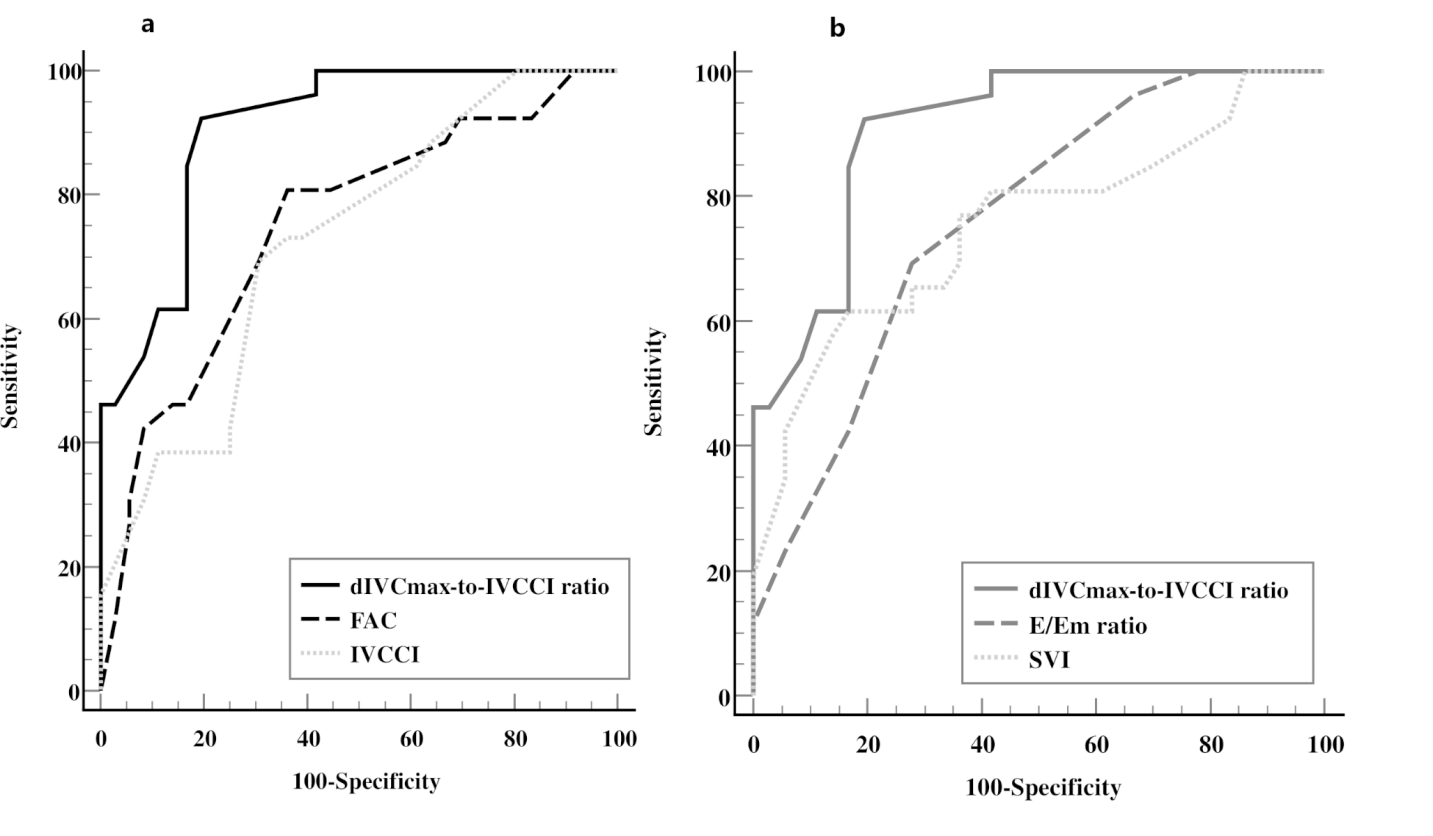
Diagnostic performance of dIVCmax-to-IVCCI ratio (ROC-AUC) with respect to (a) right ventricle (FAC and IVCCI) and (b) left ventricle (SVI and E/Em ratio) echocardiographic variables, respectively. IVCCI = inferior vena cava collapsibility index, dIVCmax = maximum diameter of IVC at expiration, FAC = fractional area change, SVI = stroke volume index, E = peak velocity flow in early diastole, Em = the average of peak velocities in early diastole of lateral and septal mitral annulus

**Fig. 4:**
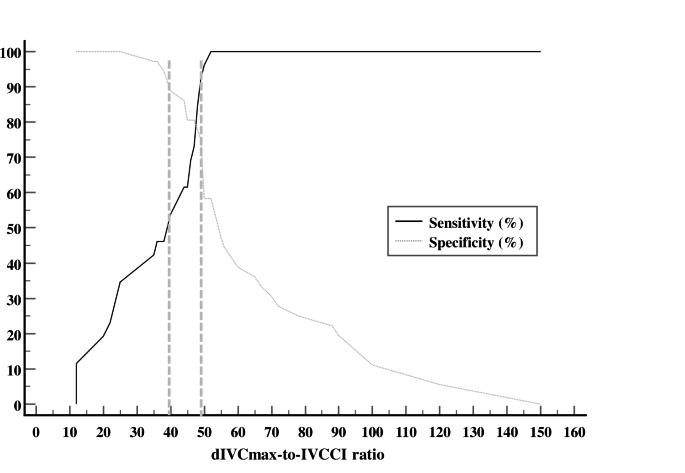
Plots of sensitivity and specificity showing the predictive ability and gray zones of the dIVCmax-to-IVCCI ratio. Gray zone plot is created using the sensitivity and specificity of the dIVCmax-to-IVCCI ratio to predict post-spinal hypotension (y-axis) against the dIVCmax-to-IVCCI ratio measurements (x-axis). The gray zone was delimited between the 90% sensitivity and the 90% specificity cutoff points on the two sigma curves. IVCCI = inferior vena cava collapsibility index, dIVCmax = maximum diameter of IVC at expiration

Preoperative dIVCmax-to-IVCCI ratio < 48 demonstrated the greatest diagnostic performance in predicting spinal-induced hypotension among various echocardiographic variables (Table 3). More specifically, the dIVCmax-to-IVCCI ratio showed higher diagnostic power with respect to the RV (IVCCI > 0.28 and FAC > 42) (Fig. 3a) and LV (E/Em ratio < 9 and SVI < 32) echocardiographic measurements (Fig. 3b). The gray zone for the dIVCmax-to-IVCCI ratio ranged between 40 and 49, included 12 (19%) cases (Fig. 4) and showed the lowest number of inconclusive measurements among the echocardiographic variables. The gray zones for FAC, IVCCI, SVI and E/Em ratio lay between 38 and 47%, 0.2–0.4, 32–44 ml/m^2^ and 7.5–10.5 cm/sec and included 32 (51.6%), 31(51.3%), 26 (41%) and 24 (39%) cases, respectively.

**Table 3:** Diagnostic performance (AUC-ROC) of baseline echocardiographic parameters in predicting spinal-induced hypotension

Data	AUC (95% CI^e^)	Cut-off value	(%) Sp	(%) Se	(%) PPV	(%) NPV
dIVCmax-to- IVCCI	0.9 (0.8–0.96) ^*, +^	< 48	80	92	78	93
IVCCI	0.72 (0.59–0.83)	> 0.28	70	69	62	75
FAC	0.74 (0.62–0.85)	> 42	81	64	62	82
SVI	0.75 (0.62–0.86)	< 32	83	61	72	75
E/Em	0.76 (0.65–0.88)	< 9	75	70	64	76

dIVCmax = maximum diameter of inferior vena cava; *IVCCI =* collapsibility index of inferior vena cava; SVI = stroke volume index; AUC = area under the curve; CI = confidence interval; Se sensitivity; Sp specificity; PPV positive predictive value; NPV negative predictive value. *Denotes statistical difference VS FAC, E/Em ratio, and SVI, *p* < 0.05. + Denotes statistical difference Vs IVCCI, *p* < 0.01

### Interobserver agreement of echocardiographic variables

**Fig. 5:**
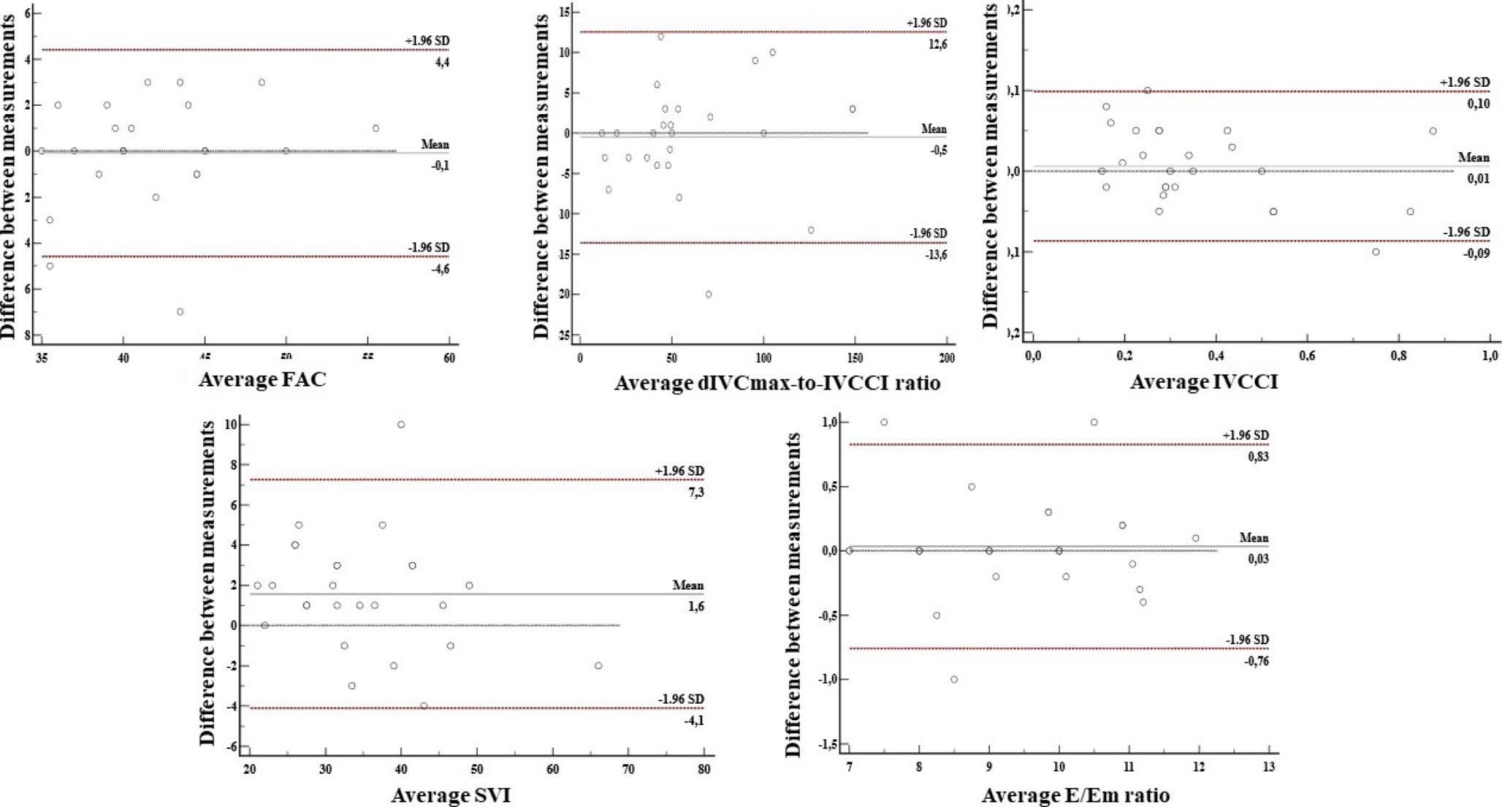
Interobserver agreement of various echocardiographic variables. IVCCI = inferior vena cava collapsibility index, dIVCmax = maximum diameter of IVC at expiration, FAC = fractional area change, SVI = stroke volume index, E = peak velocity flow in early diastole, Em = the average of peak velocities in early diastole of lateral and septal mitral annulus

Interobserver agreement was substantial for IVCCI (CCC = 0.97), dIVCmax-to-IVCCI (CCC = 0.961), E/Em ratio (CCC = 0.962) and SVI (CCC = 0.953) and moderate for FAC (CCC = 0.904). Interobserver agreement was furthered analyzed by Bland-Altman plots (Fig. 5).

### LOS in the PACU

There was a significant difference in LOS in the PACU between normotensive patients and hypotensive patients (Table 1). Among the hypotensive patients (n = 26), 22 displayed dIVCmax-to-IVCCI ratio < 48, with 9 of whom (40%) also experiencing hypotension in the PACU; the 4 hypotensive patients with dIVCmax-to-IVCCI ratio > 48 did not present hemodynamic instability in the PACU.

## Discussion

LV with reduced EF may present with a different response to spinal anesthesia when compared to normal hearts, since their ability to compensate for reduced SVR and/or preload is blunted [24–28]. Spinal anesthesia can compromise the sympathetic nervous system tone [27–30], which may affect compensatory hemodynamic functions. In our study, hypotensive patients showed higher preoperative SVR than normotensive patients, while in both groups blood pressure measurements were nearly the same at baseline. It is likely that in hypotensive patients, the sympathetic nervous system was actively compensating for low preload (as expressed by the reduced dIVCmax-to-IVCCI ratio) and its blunting due to spinal anesthesia might have led to hemodynamic decompensation, and consequently to hypotension. For this reason, the patients with increased SVR values seemed to be more prone to post-spinal anesthesia hypotension.

Hypotensive patients also showed lower E/Em ratio values with respect to normotensive patients. Additionally, the E/Em ratio < 9 exhibited sufficient diagnostic performance to predict post-spinal anesthesia hypotension and was slightly superior in this regard to the SVI. The E/Em ratio has been suggested as a reliable estimate of LV diastolic pressures [17]. Therefore, it is highly likely that the reduced venous return (due to sympathectomy) in our patients might have led to decreased LV diastolic pressures and SVI, and subsequently, to hypotension after spinal anesthesia induction.

Assessment of right ventricular function is important, but it is not easy to achieve due to the complex geometry of the RV [30]. SV of the RV is mainly generated by longitudinal shortening: the reduction of the cavity diameter (radial thickening), however, also contributes to the global function of the RV. The TAPSE and TASV measurements only assess the RV longitudinal contraction. Conversely, the FAC estimates the RV global function, including both the RV longitudinal and radial function [15]. In our study, the longitudinal contraction (TAPSE and TASV measurements) of the RV did not differ in hypotensive and normotensive patient. Nevertheless, the radial function, as partially expressed by the FAC, was substantially higher in the hypotensive patients. This may be attributed to the fact that the enhanced radial function of the RV is very likely compensatory to low preload conditions, as evidenced by the low dIVCmax-to-IVCCI ratio.

In our study, both the dIVCmax-to-IVCCI ratio and IVCCI differed between hypotensive and normotensive patients. The dIVCmax-to-IVCCI ratio has exhibited better precision in the prediction of cardiovascular hemodynamics than IVCCI alone. This may indicate that the initial reference diameter (dIVCmax) of the IVC (and its subsequent fluctuations from baseline measurements) is the most decisive factor in determining hypotension after spinal anesthesia in elderly patients with reduced LV-EF, and not the respiratory fluctuation of the IVC per se. Furthermore, given that the proportion of results in the gray zone is critical to test utility [23]; the dIVCmax-to-IVCCI ratio exhibited the lowest percentage of patients in the gray zone with respect to the IVCCI, FAC, E/Em ratio and SVI.

Rocke et al. [6] have demonstrated that spinal anesthesia in elderly males with cardiac disease substantially reduced mean arterial pressure due to decreases in SVR. Although these hemodynamic changes seemed to be well tolerated by the subjects, the post – spinal anesthesia hypotension was of sufficient magnitude that most patients needed treatment. However, this study was a small cohort, with most of patients (mean age: 67.3 ± 6.2 years) presenting LV-EF greater than 50%. Additionally, as the prevalence of chronic heart failure is increasing, more patients with chronic heart failure will require noncardiac surgery. Lee et al. [7] and Kumar et al. [8] have reported perioperative cardiac event rates of 5–10% in patients with a history of chronic heart failure subjected to noncardiac operations. Therefore, it would be clinically significant to define an optimal method for the prediction and treatment of post-spinal anesthesia hypotension in elderly patients (> 70 years) with cardiac disease and reduced LV-EF. In fact, the dIVCmax-to-IVCCI ratio seems to be very promising for the prediction of post-spinal anesthesia hypotension as it consolidates both the static (dIVCmax) and the dynamic component (IVCCI) of the IVC measurements [11, 14].

In our study, we wanted to ensure appropriate documentation of the maximum upper extent of sensory blockade. Administration of 15 mg of intrathecal plain ropivacaine in patients undergoing lower limb surgery usually reaches the T9 level, with an onset of 5 to 60 min and two dermatomes regression time at 270 min [31]. For this reason, in our study, blood pressure measurements were obtained from the induction of spinal anesthesia to the end of surgery.

Our study has limitations. We provided spinal anesthesia to a vulnerable group of patients, which may have presented an increased risk to this population. Indeed, in this subset of patients, spinal anesthesia did cause hemodynamic perturbations; however, the patients who experienced hypotension were successfully resuscitated. We considered, therefore, that spinal anesthesia might be an option in patients with mild or moderate reduction of LV-EF in whom the cardiovascular function was in a compensated status. In addition, to homogenize our patients’ sample, we did not include patients with very low LV-EF (< 35%). Patients with LV-EF < 35% represent a very vulnerable population to whom post-spinal anesthesia hypotension is a great risk. Further research on this topic is essential.

We did not enroll patients who had atrial fibrillation or left bundle branch block during the assessment. In fact, this subset of patients could have functioned as a confounding factor, making the accurate calculation of LV-EF and other echocardiographic parameters difficult [14–17].

An additional, minor shortcoming is that, although we tested multiple echocardiographic measurements in one cohort, a multiple comparisons correction test was not applied to address this multiple testing problem; however, it is considered that this drawback is not vexing and its impact on our results as well as on the interpretation of our data is not crucial.

Even though co-loading (infusion of fluids immediately after the onset of spinal anesthesia) leads to an increased hemodynamic stability [32], we decided against this to better evaluate the hemodynamic response to spinal anesthesia with minimal effect on preload and afterload. Also, we considered that the administration of unnecessary fluids may lead to complications especially in patients with LV dysfunction [33, 34]. Therefore, we would hypothesize that in patients with cardiac dysfunction and dIVCmax-to-IVCCI ratio < 49, post - spinal fluid administration could be potentially beneficial by diminishing the incidence of hypotension, while in patients with dIVCmax-to-IVCCI ratio > 49, the excessive provision of fluids could be avoided, as it leads to overhydration. Additional investigations will be required to confirm this hypothesis.

While IVC measurements have been extensively applied to predict fluid/volume responsiveness (prediction of cardiac output) in hemodynamically unstable patients under mechanical ventilation [9], our study investigated into the role of various echocardiographic measurements in the prediction of hypotension after spinal anesthesia in spontaneously breathing patients.

Furthermore, there is no single definition of intraoperative hypotension. In fact, a systematic review found 140 different definitions for intraoperative hypotension in 130 studies; all definitions were based on systolic arterial pressure, MAP values, absolute values or relative changes or a combination of them [35]. More recently, Salmasy et al. [36] demonstrated an increasing postoperative risk for longer exposure beneath certain MAP thresholds (absolute reference threshold of 65 mmHg and a relative reference threshold of 20% below baseline). They further advocated that low intraoperative blood pressure values are associated with both postoperative kidney and myocardial injury. A caveat, nonetheless, is that the authors evaluated only two organs. The patient characteristics as well as the statistical analysis suggest that this study included a highly heterogeneous group of populations. Also, in contrast to many previous studies that used preinduction MAP as “baseline value,” the authors defined baseline MAP as “average of all MAP readings (taken by sphygmomanometer) over 6 months period before surgery, excluding measurements during a hospital stay. In our study, we decided to define intraoperative hypotension as an absolute MAP ≤ 65 mmHg or a reduction ≥ 25% of baseline preoperative values because an absolute threshold of MAP ≤ 65 mmHg or a 20–30% (average 25%) reduction of blood pressure below preoperative reference values are frequently used to define intraoperative hypotension events [35, 37].

Continuous invasive blood pressure monitoring through radial artery line placement can be difficult, painful, and time consuming thereby causing significant distress to the patient [38, 39]. Under these circumstances, baseline blood pressure measurements may be falsely elevated. In view of these factors, we assumed that preoperative non-invasive baseline blood pressure assessment with a sphygmomanometer may be closer to the “real” daily life blood pressure measurements of the patients [36]. However, in the intraoperative period, continuous invasive blood pressure measurements facilitate the early detection of intraoperative hemodynamic perturbations, whereas taking frequent blood pressure measurements using a sphygmomanometer (every one to three minutes) may induce patient discomfort, increase the risk of skin and nerve damage, as well as causing a delay in detection of significant hemodynamic perturbations [40, 41]. Also, the endpoint of our study was hypotensive episodes lasted for only 30 s, which would be difficult, however, to be recorded by using a sphygmomanometer. We did not consider hypotension events for longer intervals since it is very well documented that postoperative complications according to hypotension are time dependent [36, 37]. Additionally, this variation has great importance when observational studies report relationships between hypotension and adverse postoperative outcomes [36, 37] which, in fact, was not the scope of our study. Therefore, we decided to exploit the advantages of these two different methods of blood pressure measurements (non-invasive blood pressure measurements prior to induction of spinal anesthesia and invasive blood pressure measurements after its induction). Nevertheless, we are aware that pertinent studies that compare the two methods of monitoring of blood pressure in elderly orthopedic patients under spinal anesthesia have yet to be published. Furthermore, our method is feasible only in a selected group of patients (elderly-trauma patients undergoing spinal anesthesia with ropivacaine).

## Conclusion

The dIVCmax-to-IVCCI index demonstrated the greatest diagnostic utility for the prediction of post - spinal anesthesia hypotension in elderly patients with LV dysfunction. This index may assist in stratyfying patients, who are at risk for intraoperative hypotension, as well as may conduce to therapeutic interventions (i.e., volume expansion) in the future.

## Data Availability

The data that support the findings of this work are available from the corresponding author upon reasonable request.
